# Association of driving while intoxicated and suicide ideation and attempts in South Korea: a study in a nationally representative sample

**DOI:** 10.1038/s41598-023-40829-8

**Published:** 2023-08-30

**Authors:** Namwoo Kim, Jieun Lee, Bong-Jin Hahm, Bo Ram Yang

**Affiliations:** 1 Department of Psychiatry, Seoul St. Mary’s Hospital, The Catholic University of Korea, College of Medicine, Seoul, Republic of Korea; 2 Department of Pediatrics, Inje University College of Medicine, Ilsan Paik Hospital, Goyang, Republic of Korea; 3 Department of Neuropsychiatry, Seoul National University Hospital, Seoul, Republic of Korea; 4 Department of Psychiatry and Behavioral Sciences, Seoul National University College of Medicine, Seoul, Republic of Korea; 5 College of Pharmacy, Chungnam National University, Daejeon, Republic of Korea

**Keywords:** Medical research, Epidemiology

## Abstract

Evidence indicating driving as a means for suicide has been consistently reported. However, there have been few reported studies examining the association between driving while intoxicated (DWI) and suicide. We aimed to investigate the associations between DWI and suicide ideation and suicide attempts taking into account the frequency and amount of alcohol consumption. This cross-sectional study used data from semi-structured, face-to-face interviews conducted with a nationally representative sample of the Korea Community Health Survey in 2009, 2013, and 2017. The study included 267,457 adult participants who drank alcohol at least once and had driven a motor vehicle at least once in the preceding year. The DWI group comprised participants who had driven under the influence of alcohol at least once in the preceding year. The associations between DWI and suicide ideation and suicide attempts were examined using a logistic regression model, adjusting for the frequency and amount of alcohol consumption and history of depressive episodes in the preceding year. The role of DWI as a risk factor for suicide was also examined in an analysis stratified by the frequency and amount of alcohol consumption. The DWI group included 39,062 (14.6%) subjects, who were at higher risk for suicide ideation (adjusted odds ratio [aOR] = 1.91; 95% CI 1.81–2.01) and suicide attempts (aOR = 1.56; 1.27–1.92) than those not involved in DWI. Increased risks of suicide ideation and suicide attempts were observed in most strata in the stratified analysis. People who engage in DWI may have an increased risk of suicide ideation and suicide attempts; this relationship was generally observed regardless of the frequency and amount of alcohol consumption.

## Introduction

In 2021, a study conducted in the United States revealed that one million people are arrested for driving while intoxicated (DWI) every year, and out of these individuals, one-third are repeat offenders^1^. Another study, based on self-reported data, estimated that there were 505 instances of DWI per 1000 people annually^2^. In 2020, alcohol-related deaths accounted for 30.0% of all driving-related deaths in the United States^3^, and 9.3% in South Korea^4^. As such, DWI is a significant social problem that can cause harm to both individuals and others^5^.

Several factors have been identified as risk factors for DWI, including depressive disorders, alcohol use disorder, impulsivity, sensation seeking, low risk avoidance, and aggressiveness^6^. Among these factors, alcohol use disorder and depressive disorders are also well-known risk factors for suicide^7^. Therefore, it can be speculated that DWI may be associated with suicide ideation or suicide attempts.

Suicide is typically a conscious and intentional act, such as hanging or shooting oneself, but there may also be unconscious suicide attempts that the person is unaware of^8^. It has been suggested that driving may also be a conscious or unconscious means of attempting suicide^9^. In a retrospective autopsy study, 9% of 406 driver fatalities were suspected suicides^10^. Individuals with suicide ideation were four times more likely to experience a car crash^11^, and 14.8% of those with a suicide plan considered using a car crash as a means^12^. In light of this research background, it is possible to consider that DWI may be an expression of suicide ideation or a method of a conscious or unconscious suicide attempt.

However, studies on the association between DWI and suicide are very rare. One study examined the association between suicide and government policy for regulating DWI^13^. The study found that the presence of zero tolerance laws, which prohibit drivers under the legal drinking age from DWI, was associated with a 10.3% decline in suicides among males aged 15 to 17. Although the regulation of DWI may have led to a decrease in suicide, the authors reported limitations in interpretation that the law may have reduced suicide by reducing drinking itself. Moreover, although DWI and suicide share risk factors related to alcohol use, it is not known whether there is an association between DWI and suicide in people who do not binge drink or who are not dependent on alcohol.

The capacity of a DWI crash, possibly with suicidal intent, to cause individual and collateral harm to others, renders it a public health issue that warrants research. In this study, a nationwide survey of a representative sample of Koreans conducted by the government was used to investigate the association between DWI and suicide ideation and suicide attempts and to determine if there was a difference in the association depending on the frequency and amount of alcohol consumption.

## Methods

### Study design and participants

This study retrospectively analyzed data from the Korea Community Health Survey (KCHS) which included a nationally representative sample^14^. The KCHS, community-based cross-sectional survey, has been conducted annually at national public health centers since 2008 by the Korea Centers for Disease Control and Prevention. It investigates health-related information of adults concerning health behaviors, disease prevalence, the utilization of medical services, and lifestyle. Based on the adults aged 19 years or older who registered as South Korean, the community (Tong∙Ban/Lee) was selected by the probability proportionate sampling method, and then the survey households were selected via systematic sampling. The sampling process was performed independently each year. A trained interviewer visits the selected study participants’ households and conducts semi-structured, face-to-face interviews, and approximately 200,000 people participate in the survey each year. As participants’ information was deidentified, matching individuals across different annual database were not available. Data from the KCHS can be obtained by requesting the use of the data for research purposes. We used the survey results from 2009, 2013, and 2017, which included surveys on DWI and mental health with information related to suicide. We only used data from 3 years—2009, 2013, and 2017—because suicide-related questionnaires were not included in the survey every year, but rather every 4 years. Participants who had consumed alcohol at least once in the preceding year and had driven a vehicle at least once in the preceding year were included in our study. The group classified as DWI was determined by those who answered “yes” to the question regarding whether they had engaged in at least one instance of DWI in the preceding year. On the other hand, the control group was defined as those who responded “no” to the same question, indicating that they had no experience with DWI in the preceding year.

The Institutional Review Board of Seoul National University Hospital and the Seoul National University College of Medicine approved this study protocol and waived the requirement for written informed consent. In addition, all personal information was anonymized in this study and all methods were performed in accordance with the relevant guidelines and regulations.

### Measures

#### Alcohol-related measures

Alcohol use was measured with a Likert scale for both the average frequency and amount of alcohol consumption during the preceding year. The unit of the amount of alcohol consumed at a time was a drink, and it referred to as either a can or bottle of beer, a glass of wine or champagne, a shot or jigger of liquor, or a mixed drink. The frequency of alcohol consumption was classified as infrequent (once a month or less), moderate (2–4 times a month), and frequent (2 times a week or more), and the amount of alcohol consumed at one time was classified as light (1–4 drinks), moderate (5–6 drinks), and heavy (7 or more drinks).

#### Mental health measures

To measure the respondents’ mental health outcome, the subjects were asked separate questions about whether they had suicide ideation at least once in the preceding year or suicide attempts at least once in the preceding year. In addition, subjects were asked if they had felt sad or hopeless enough to have difficulty carrying out their daily routine for two or more weeks continuously in the preceding year: we defined this as a depressive episode. Since depression is known as a risk factor for both DWI and suicide^15^, the depressive episode was included in the baseline characteristics.

#### Demographic measures

Demographic variables included age, sex, monthly income, Medical AID recipient status, household type, area of residence, occupation type, employer/employee status, education level, marital status, and smoking status were investigated. Those who received Medical AID were individuals who were unable to pay for national health insurance coverage. Monthly income was divided into 4 categories based on 1, 3, and 5 million KRW (1 million KRW = approximately 1000 USD).

#### Driving-related measures

For the driving-related variables, whether the subjects routinely used seat belts was measured using a 5-point Likert scale, and if a participant answered “I usually wear it” or “I always wear it,” he or she was considered to wear a seat belt^2,16^. Whether the participants rode in a vehicle operated by another person with DWI was also measured. Furthermore, we investigated the number of instances of DWI in the preceding year. The questions regarding the number of instances of DWI were included in the surveys conducted in 2009 and 2013, but not in 2017. Thus, this study only utilized data from the 2009 and 2013 surveys.

Detailed information regarding the survey questions conducted by KCHS can be found in the Supplementary Tables 1 and 2.

#### Exposure and outcomes

The exposure was DWI. The primary outcome was whether DWI was associated with suicide ideation and whether DWI was associated with suicide attempts. The secondary outcomes were (1) the difference in the risk of suicide ideation and attempts based on the number of instances of DWI, and (2) the result of a stratified analysis in which the primary analysis was repeated for each frequency of alcohol consumption and amount of alcohol consumption stratum. Stratified analyses for the other variables were also conducted.

### Statistical analysis

Frequencies for categorical variables and mean values for continuous variables were used to indicate the characteristics of the subjects. We used *t*-test and chi-square test to compare the distribution between the DWI group and the control group according to the variables.

To examine the associations between DWI and suicide ideation and suicide attempts, odds ratios (ORs) and 95% confidence intervals (CIs) were calculated using a logistic regression model. Adjusted odds ratios (aORs) and 95% CIs were estimated after adjusting for the following covariates: age, sex, monthly income, Medical AID recipient status, household type, area of residence, occupation type, employer/employee status, education level, marital status, smoking status, frequency of alcohol consumption, amount of alcohol consumed at a time and depressive episode in the preceding year. In the analysis of the association between the number of instances of DWI and suicide ideation and attempts, the analysis was divided into two groups: the first analysis included both the control group and the DWI group, and the second analysis was conducted only on the DWI group. We also conducted a homogeneity test to examine the difference in the associations between DWI and suicide ideation and suicide attempts across strata of frequency and amount of alcohol consumption. Statistical analyses were performed using SAS version 9.4. A two-sided *p-*value < 0.05 was considered to indicate statistical significance.

## Results

**Figure 1:**
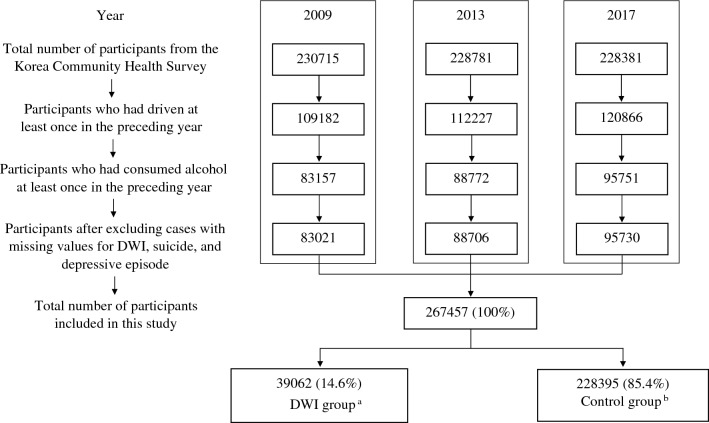
Study flow. ^a^People who had driven a motor vehicle under the influence of alcohol at least once in the preceding year. ^b^People who had driven a motor vehicle at least once and consumed alcohol at least once in the preceding year, but had not driven under the influence of alcohol.

In 2009, 2013, and 2017, the number of respondents to the survey was about 220,000 to 230,000 each year, and a total of 267,457 people who had consumed alcohol at least once and had driven at least once in the preceding year were included in the present study (Fig. 1). Among them, 39,062 (14.6%) were in the DWI group and 228,395 (85.4%) were in the control group.

All variables included in the baseline characteristics were significantly different between the groups (Table 1). The participants in both groups were aged in their mid-40s on average (46.5 ± 11.8 in the DWI group and 44.9 ± 12.4 in the control group, *p* < 0.001), and the proportion of men was higher in the DWI group (85.0%) than in the control group (66.0%, *p* < 0.001). Compared to the control group, the DWI group had a lower proportion of metropolitan dwellers (21.4% vs. 32.3%, *p* < 0.001) and unemployed people (9.7% vs. 19.7%, *p* < 0.001). More subjects in the DWI group suffered depressive episode in the preceding year than in the control group (6.47% vs. 4.53%, *p* < 0.001). There were significant differences between the groups in both the amount (*p* < 0.001) and frequency (*p* < 0.001) of drinking. In the DWI group, fewer of the subjects answered that they wore seat belts (79.4% vs. 93.0%, *p* < 0.001) than in the control group.

**Table 1:** Baseline characteristics.

	DWI group (%)	Control group (%)	*p* value
Total number of participants	39,062	228,395	
Age, mean (SD)	46.5 ± 11.8	44.9 ± 12.4	< 0.001
19–39	11,616 (29.7)	82,441 (36.1)	< 0.001
40–64	24,496 (62.7)	130,019 (56.9)	
65+	2950 (7.6)	15,935 (7.0)	
Sex			
Male	33,218 (85.0)	150,820 (66.0)	< 0.001
Female	5844 (15.0)	77,575 (34.0)	
Year			
2009	15,786 (40.4)	67,235 (29.4)	< 0.001
2013	12,061 (30.9)	76,645 (33.6)	
2017	11,215 (28.7)	84,515 (37.0)	
Monthly income			
< 1	3184 (8.9)	16,295 (7.8)	< 0.001
1–3	14,347 (39.9)	74,017 (35.3)	
3–5	12,656 (35.2)	79,184 (37.7)	
> 5	5791 (16.1)	40,392 (19.2)	
Medical AID recipient	424 (1.1)	2187 (1.0)	0.017
Household type			
Single-person households	3133 (8.0)	15,588 (6.8)	< 0.001
Others	35,928 (92.0)	212,786 (93.2)	
Area of residence			
Metropolitan	8358 (21.4)	73,873 (32.3)	< 0.001
Others	30,704 (78.6)	154,522 (67.7)	
Occupation type			
Unemployed	3798 (9.7)	45,060 (19.7)	< 0.001
Clerks	11,480 (29.4)	73,141 (32.0)	
Service workers	13,588 (34.8)	76,608 (33.5)	
Agricultural workers	7316 (18.7)	17,675 (7.7)	
Elementary workers	2880 (7.4)	15,911 (7.0)	
Employer/employee			
Employer	15,283 (44.6)	56,709 (32.2)	< 0.001
Employee	18,982 (55.4)	119,630 (67.8)	
Education level (years)			
≤ 9	7232 (18.5)	30,774 (13.5)	< 0.001
10–12	15,515 (39.8)	82,841 (36.3)	
≥ 13	16,261 (41.7)	114,469 (50.2)	
Marital status			
Living with a partner	30,421 (78.0)	174,083 (76.3)	< 0.001
Divorce/legal separation/widowed	3385 (8.7)	17,174 (7.5)	
Never married	5217 (13.4)	36,939 (16.2)	
Smoking status			
Current smoker	17,352 (44.5)	72,025 (31.5)	< 0.001
Ex-smoker	11,130 (28.5)	48,196 (21.1)	
Non-smoker	10,556 (27.0)	108,106 (47.4)	
Depressive episode			
Yes	2529 (6.47)	10,340 (4.53)	< 0.001
No	36,533 (93.53)	218,055 (95.47)	
Frequency of alcohol consumption			
Infrequent (≤ once/month)	4029 (10.32)	75,034 (32.86)	< 0.001
Moderate (2–4 times/month)	11,454 (29.33)	77,047 (33.74)	
Frequent (≥ 2 times/week)	23,575 (60.36)	76,251 (33.39)	
Amount of alcohol consumed at a time (drink)			
Light (1–4)	11,561 (29.6)	117,343 (51.4)	< 0.001
Moderate (5–6)	6725 (17.22)	34,298 (15.02)	
Heavy (≥ 7)	20,770 (53.18)	76,669 (33.58)	
Seat belt use as a driver			
Yes	30,994 (79.4)	212,328 (93.0)	< 0.001
No	8062 (20.6)	16,031 (7.0)	
Seat belt use as a front passenger			
Yes	23,874 (72.3)	165,343 (87.8)	< 0.001
No	9171 (27.8)	22,932 (12.2)	
Experience as a passenger of a person engaged in DWI			
Yes	15,645 (40.1)	16,381 (7.2)	< 0.001
No	23,378 (59.9)	211,959 (92.8)	

*DWI* Driving While Intoxicated.

Odds ratios of suicide ideation and suicide attempts are shown in Table 2. Individuals with DWI had a 1.91 (1.81–2.01) and 1.56 (1.27–1.92) times greater likelihood of suicide ideation and suicide attempts compared to individuals without DWI, respectively.

**Table 2:** Odds ratios of main outcomes.

	No. of event (%)	Crude OR (95% CI)	Adjusted OR (95% CI)^a^
Suicide ideation			
DWI group	3820 (9.8)	1.85 (1.78–1.92)	1.91 (1.81–2.01)
Control group	12,630 (5.5)	1 (ref)	1 (ref)
Suicide attempt			
DWI group	210 (0.54)	2.03 (1.73–2.37)	1.56 (1.27–1.92)
Control group	607 (0.27)	1 (ref)	1 (ref)

*DWI* Driving While Intoxicated.

^a^Adjusted for the variables including age, sex, income, medical AID recipient status, types of households, region of residence, types of occupations, education level, marital status, smoking status, frequency of alcohol consumption, amount of alcohol consumption, and depressive episode in the preceding year.

Table 3 presents the results of an analysis examining the association between the number of instances of DWI and the risk of suicide ideation and suicide attempts. Both the total and DWI groups, the risk of suicide ideation increased significantly as the number of instances of DWI increased in both 2009 and 2013. In the case of suicide attempts, both groups demonstrated a significant increase in the risk of suicide attempts as the number of instances of DWI increased in 2009, while in 2013, neither group showed significance.

**Table 3:** Odds ratios of outcomes according to the number of instances of DWI.

	2009		2013	
	Crude OR (95% CI)	Adjusted OR^b^ (95% CI)	Crude OR (95% CI)	Adjusted OR^b^ (95% CI)
Suicide ideation				
Total group^a^	1.008 (1.006–1.009)	1.006 (1.004–1.008)	1.008 (1.006–1.01)	1.007 (1.005–1.009)
DWI group	1.004 (1.002–1.006)	1.004 (1.002–1.006)	1.004 (1.002–1.006)	1.004 (1.002–1.007)
Suicide attempt				
Total group^a^	1.009 (1.006–1.013)	1.007 (1.002–1.011)	1.004 (0.995–1.014)	0.996 (0.976–1.016)
DWI group	1.007 (1.004–1.011)	1.007 (1.001–1.012)	1.000 (0.987–1.013)	0.989 (0.959–1.021)

*DWI* Driving While Intoxicated.

^a^Total group refers to the entire group including both the control group and the DWI group.

^b^Adjusted for the variables including age, sex, income, medical AID recipient status, types of households, region of residence, types of occupations, education level, marital status, smoking status, frequency of alcohol consumption, amount of alcohol consumption, and depressive episode in the preceding year.

Table 4 shows the results of the stratified analyses of the frequency and the amount of alcohol consumption. The aORs of suicide ideation were significantly greater than 1 for all strata. With respect to the frequency of alcohol consumption among the subjects who drank at moderate or frequent level, the aORs of suicide attempts were significantly greater than 1. However, for infrequent drinking, the trend did not reach statistical significance (*p* = 0.052). With respect to the amount of drinking for the subjects who drank at light and moderate level, the aORs of suicide attempts were significantly greater than 1. However, in the case of heavy level, there was no significant difference (*p* = 0.93). Homogeneity test results showed that the risk of suicide ideation and suicide attempts does not significantly vary across frequency and amount strata (Supplementary Table 3).

**Table 4:** Stratification analyses of frequency and amount of alcohol consumption.

	Crude OR (95% CI)	*p* value	Adjusted OR (95% CI)	*p* value
Frequency of alcohol consumption				
Infrequent (≤ once/month)				
Suicide ideation	2.05 (1.86–2.27)	< 0.001	1.95 (1.70–2.24)	< 0.001
Suicide attempt	2.34 (1.55–3.52)	< 0.001	1.74 (1.00–3.05)	0.052
Moderate (2–4 times/month)				
Suicide ideation	1.97 (1.83–2.12)	< 0.001	2.02 (1.83–2.22)	< 0.001
Suicide attempt	2.05 (1.46–2.87)	< 0.001	1.81 (1.18–2.78)	0.006
Frequent (≥ 2 times/week)				
Suicide ideation	1.85 (1.75–1.95)	< 0.001	1.88 (1.76–2.01)	< 0.001
Suicide attempt	1.79 (1.45–2.20)	< 0.001	1.53 (1.18–1.98)	0.001
Amount of alcohol consumed at a time (drink)				
Light (1–4)				
Suicide ideation	1.82 (1.70–1.94)	< 0.001	1.98 (1.81–2.17)	< 0.001
Suicide attempt	2.03 (1.52–2.70)	< 0.001	1.63 (1.10–2.39)	0.014
Moderate (5–6)				
Suicide ideation	2.02 (1.83–2.23)	< 0.001	2.06 (1.81–2.34)	< 0.001
Suicide attempt	2.49 (1.64–3.75)	< 0.001	2.11 (1.25–3.56)	0.005
Heavy (≥ 7)				
Suicide ideation	1.98 (1.79–2.19)	< 0.001	1.80 (1.59–2.04)	< 0.001
Suicide attempt	1.70 (1.13–2.55)	0.011	1.02 (0.61–1.72)	0.93

**Figure 2:**
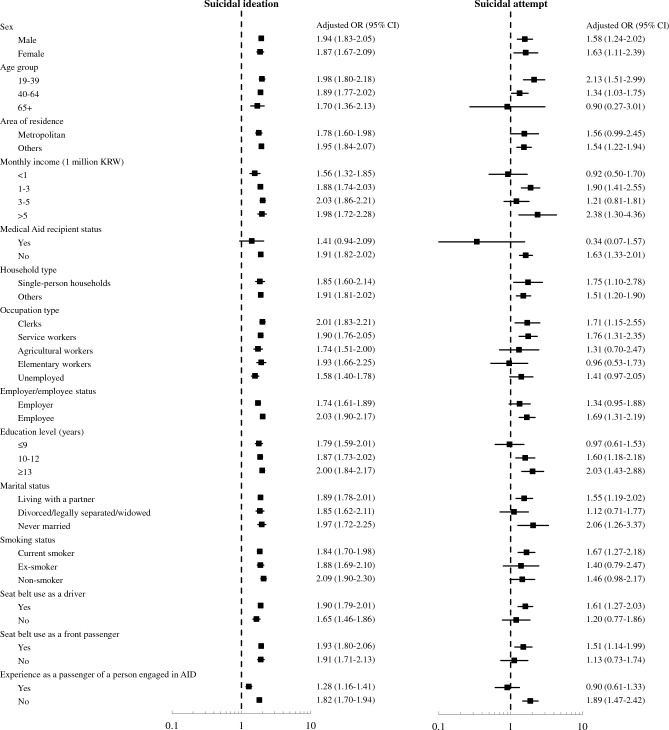
Forest plot of the stratified analysis. *DWI* Driving While Intoxicated.

The results of the stratified analyses for other variables are shown in Fig. 2. Increased risk of suicide ideation was observed in most strata in the stratified analysis. For the risk of suicide attempt, the aORs were significantly greater than 1 in several strata, including male, female, high-income, highly educated, and current smokers.

## Discussion

This study investigated the risk of suicide ideation and suicide attempts among with DWI using data from a nationwide survey of a representative sample. About 1 in 7 of the subjects (14.6%) had engaged in DWI at least once in the preceding year, and there were differences between the DWI group and the control group related to various socio-demographic characteristics, history of depressive episodes and the frequency and amount of alcohol consumption. Even after adjusting for the history of depressive episodes, the frequency and amount of alcohol consumption, the risks of suicide ideation and suicide attempts were higher in the DWI group. Moreover, as the number of instances of DWI increased, the risk of suicide ideation increased, while the risk of suicide attempts demonstrated varying results depending on the year. The stratified analysis demonstrated that individuals with a history of DWI exhibited a higher risk of suicide ideation across all categories of alcohol consumption in terms of both frequency and amount of drinking. In addition, the risk of suicide attempts associated with DWI was generally significant, with the exception of two strata. According to the homogeneity test, the odds ratios of suicide ideation and suicide attempts exhibited no significant differences across strata of frequency and amount of drinking.

Possible explanations for the association between DWI and suicide are as follows. First, certain personality types or temperaments can mediate the risk of DWI and suicide. Characteristics such as low social responsiveness, lack of self-control, hostility, poor decision-making, aggression, impulsivity, and antisocial personality disorder have been found to be representative risk factors for DWI, occurring even more frequently among those who had engaged in DWI multiple times^15^. Some of these risk factors associated with DWI are known to be risk factors for suicide as well. Antisocial personality disorder has also been found in previous studies to be associated with the risk of suicide^17,18^. Sensation seeking has been found to be an independent predictor of DWI as a risk-taking behavior pattern and increase the risk of suicide among adolescents^15,19,20^.

Second, the association between DWI and suicide may be mediated by depression. Depression is the most common psychiatric illness among people who die from suicide, and it is often accompanied by various cognitive impairments such as decreased judgment and self-control that can lead to excessive drinking or DWI^21–25^. Interestingly, in this study, the association between DWI and suicide was significant even after adjusting for the depressive episodes. This is a novel result, suggesting that even if people do not suffer from depression, DWI can be accompanied by an elevated risk for suicide ideation or suicide attempts.

Third, individuals who experience suicide ideation might have engaged in DWI, or they may have utilized DWI as a method for attempting suicide. Although there have been few studies examining DWI as a method of attempting suicide, an association between motor vehicle crashes and suicide has been observed. For example, studies have reported rates of motor vehicle crashes deaths involving suspected suicide of 3.1% in Sweden and 5.9% in Finland^26,27^. Some individuals contemplating suicide may also believe that suicide involving a motor vehicle crashes can hide their suicide intentions and bring economic benefits to their families, thus leading them to consider DWI as a method for attempting suicide^28^.

The intention to commit suicide can be conscious or unconscious, and the methods for attempting suicide can be direct, as well as indirect^8^. It has been suggested that motor vehicle crashes involving a single car can, in some cases, be an unconscious suicide attempt^29^. It is difficult to determine with certainty whether a motor vehicle crashes caused by DWI is an unconscious or indirect suicide attempt, but the proportion of instances in which it is an unconscious suicide attempt is likely to be underestimated^9^. While this study did not demonstrate that DWI is an unconscious suicide attempt, it highlighted the need to consider the possibility by indicating an association between DWI and suicide ideation and suicide attempts.

On the other hand, there were other studies that showed an association between DWI and suicide in a context other than DWI being interpreted as a suicide attempt. According to a study, individuals who have caused harm or fatalities to others as a result of DWI are more prone to experiencing suicidal tendencies due to overwhelming feelings of guilt^30^. Another study, focused on the elderly population, revealed that ceasing to drive due to factors such as DWI can result in a loss of independence, potentially leading to suicide^31^. The aforementioned two studies indicate that while there is a statistical association between DWI and suicide, it is crucial to approach the interpretation of DWI as a method of suicide attempt with great caution.

This study has the following limitations. First, information on DWI was evaluated based on self-reported data. However, studies have found self-reported data on driving behavior to be fairly accurate^32,33^. In this study, from 2009 to 2017, the number of subjects included in the DWI group decreased, which coincides with a decrease in the number drunk driving incidents, injuries and deaths reported by the Korea Road Traffic Authority during 2009–2017^34^. Future studies should evaluate the suicide tendencies of people who have been objectively confirmed to have engaged in DWI through a blood alcohol concentration test. Second, since this study examined the subjects’ experiences from the entire preceding year, DWI and instances of suicide ideation and suicide attempts may not have occurred at the same time, and there may have been discrepancies in time. Furthermore, due to the cross-sectional nature of the study design, causal or temporal relationships could not be determined. Third, The criteria for the depressive episode that we defined in our study differ from the DSM-5’s diagnostic criteria for a major depressive episode^35^. Moreover, as the assessments were based on self-reports, it is difficult to determine whether the severity of the participants' depressive episode reached the level of a major depressive episode. Additionally, our study did not use international diagnostic criteria for the depressive episode, making it challenging to compare our results with those of other studies. Forth, driving distance and driving frequency are known to be important variables in studies examining the relationship between driving and suicide^36^. This is because individuals who drive long distances or frequently are known to have a higher likelihood of DWI^37^. Therefore, these variables should be considered as confounding factors in the data analysis. However, the survey used in our study did not include questions about driving distance and frequency, and as a result, we were unable to collect data on these variables. Fifth, the KCHS conducted new sampling for each year, and thus did not track the same subjects longitudinally across multiple years. However, although the probability is low, there is a possibility that the same person participated in the KCHS in different years. Sixth, the frequency and amount of alcohol consumption questions used in this study do not adhere to internationally recognized standards, such as those established by the Centers for Disease Control and Prevention (CDC)^38^, National Institute on Alcohol Abuse and Alcoholism (NIAAA)^39^, or World Health Organization (WHO)^40^. As a result, the categorization of alcohol consumption, for example, light/moderate/heavy, lacks a rigorous academic foundation, which also presents challenges in comparing these findings with those of other studies. Finally, the subjects in this study were Korean, and differences in the laws and cultures of other countries may have a significant impact on the relationship between DWI and suicide. Further studies are needed to examine this association in other countries.

The important findings of this study include that DWI was associated with suicide ideation and suicide attempts, largely irrespective of drinking frequency, drinking amount and history of depressive episodes. Evidences had been reported that driving has been used as a method of suicide attempts. However, studies examining the association between DWI and suicide ideation or suicide attempts were extremely rare, so this study tried to provide an evidence for this. The possibility that DWI and suicide are likely to occur together has an important implication for medical service providers and policy-makers because people at risk of suicide can harm not only themselves, but also others as a result of a traffic crashes caused by DWI. Although effective social campaigns and laws are being introduced to reduce DWI^36,41^, more research on DWI from the perspective of mental health is needed.

### Supplementary Information

Supplementary Tables.

## Data Availability

The Korea Community Health Survey (KCHS) raw data can be downloaded and used at https://chs.kdca.go.kr/chs/index.do for research purposes, or available from the corresponding author on reasonable request.
