# Supplementary material for: Association of driving while intoxicated and suicide ideation and attempts in South Korea: a study in a nationally representative sample
Source: Sci Rep. 2023 Aug 30;13:14199. doi: 10.1038/s41598-023-40829-8 (PMC10468494; doi:10.1038/s41598-023-40829-8)
Supplement: Supplementary file 1 — Supplementary Tables. [file 41598_2023_40829_MOESM1_ESM.docx]

**Supplementary Material**

**Table 1. Detailed description of the variables used in this study**

| Variables that require further explanation | Detailed description of the variable |
| --- | --- |
| Depressive episode | We acknowledge upon further review that the original sentence regarding the definition of the depressive episode might lead to confusion. The following is what we've previously described in the manuscript.  “the subjects were asked whether they experienced suicidal ideation or had attempted suicide, or experienced a depressive mood that interfered with their daily life for at least 2 weeks (we defined this as a depressive episode) at least once during the preceding year.”  To make the meaning of the sentence clearer, we have separated the content regarding the suicide and the depressive episode. Additionally, we have explicitly described the questions used in the survey to assess the depressive episode.  “The subjects were asked separate questions about whether they had suicide ideation at least once in the preceding year or suicide attempts at least once in the preceding year. In addition, subjects were asked if they had felt sad or hopeless enough to have difficulty carrying out their daily routine for two or more weeks continuously in the preceding year: we defined this as a depressive episode.”  According to the Diagnostic and Statistical Manual of Mental Disorders-5 (DSM-5)^1^, established by the American Psychiatric Association, a major depressive episode can be summarized as the presence of depression-related symptoms (such as depressive mood, loss of interest, and suicide ideation) that persist for at least two weeks. Simultaneously, these symptoms must cause clinically significant distress or impairment in social, occupational, or other important areas of functioning. Bearing this in mind, the questions used in this study to assess depression do not evaluate a major depressive episode, nor can they be considered a professionally diagnosed major depressive episode due to their self-report nature. We have acknowledged these limitations and addressed them in the discussion section of our manuscript. Based on this, the questions used in this study to assess depression do not align with the DSM-5 diagnostic criteria for the major depressive episode. Furthermore, because the assessment was based on self-report, it cannot be referred to as a diagnosis of major depressive episode, which should be done by a mental health professional. We have described these limitations of our study in the discussion section of our manuscript.  “The criteria for the depressive episode that we defined in our study differ from the DSM-5's diagnostic criteria for a major depressive episode.^1^ Moreover, as the assessments were based on self-reports, it is difficult to determine whether the severity of the participants' depressive episode reached the level of a major depressive episode. Additionally, our study did not use international diagnostic criteria for the depressive episode, making it challenging to compare our results with those of other studies.” |
| Demographic variables:  Household type | Participants in the study answered yes/no to the question of whether they were currently living in a single-person household. |
| Demographic variables:  Medical AID repcipient | Participants in the study answered yes/no to the question of whether they were current Medical AID recipients. |
| Demographic variables:  Area of residence | Research participants submitted their current residence address, and in this study, only whether the residence was a metropolitan or not was classified. |
| Demographic variables:  Occupation type | The current occupation was asked to choose one of the following categories:  1) Unemployed, 2) Clerks, 3) Service workers, 4) Agricultural workers, 5) Elementary workers. |
| Demographic variables:  Employer/Employee | Among those who answered that they are currently working, the research participants were asked which of the following categories of work they were doing.  1) Employers and self-employed (I do my own business)  2) Wage workers (employed by another person or a company and working for remuneration)  3) Unpaid family workers (helping family members and relatives with their work without receiving money)  In this study, those who chose #1 were defined as employers, and those who chose #2 or #3 were defined as employees. |
| Demographic variables:  Education level | The question about the educational level of the research participants is to choose one of the following.  1) None, 2) Seodang, 3) Elementary school, 4) Middle school,  5) High school, 6) 2-year/3-year university, 7) 4-year university,  8) Graduate school or higher.  In this study, it was divided into three stages. Categories #1-#4 were defined as less than or equal to 9 years of education, #5 was defined as 10-12 years of education, #6-#8 were defined as equeal to or more than 13 years of education. |
| Demographic variables:  Marital status | The current mariatal status was asked to choose one of the following categories:  1) Living with a partner, 2) Divorce/Legal separation/Widowed,  3) Never married |
| Demographic variables:  Smoking status | The current smoking status was asked to choose one of the following categories:  1) Current smoker, 2) Ex-smoker, 3) Non-smoker |
| Driving-related variables:  Seat belt use as a driver | Research participants were asked the following questions:  “Do you wear a seat belt when driving?”  1) Not at all, 2) I rarely wear them, 3) Sometimes I wear it,  4) I usually wear it, 5) always wear  In this study, those who selected #4-#5 were assigned to the "yes" group of the "Seat belt use as a driver" variable. |
| Driving-related variables:  Seat belt use as a front passenger | Research participants were asked the following questions:  “Do you wear a seat belt when sitting in the front seat?”  1) Not at all, 2) I rarely wear them, 3) Sometimes I wear it,  4) I usually wear it, 5) always wear  In this study, those who selected #4-#5 were assigned to the "yes" group of the "Seat belt use as a front passenger" variable. |
| Experience as a passenger of a person engaged in DWI | Research participants answered yes/no to the following questions.  “Have you ever been in a car driven by someone who has had any alcohol in the preceding year?”  Those who answered ‘yes’ to this question were assigned to the ‘yes’ group of this variable. |

Table 2. Questions related to the frequency and quantity of drinking used in this study

|  | Original categories used in KCHS | Categories used in this study |
| --- | --- | --- |
| **Quantity:**  How much alcohol do you drink at one time? | 1) 1-2 drinks  2) 3-4 drinks  3) 5-6 drinks  4) 7-9 drinks  5) more than 10 drinks | 1) mild (1-4 drinks)  2) moderate (5-6 drinks)  3) severe (7 or more drinks) |
| **Frequency:**  How often do you drink alcohol? | 1) less than once a month  2) once a month  3) 2-4 times a month  4) 2-3 times a week  5) more than 4 times a week | 1) mild (once a month or less)  2) moderate (2-4 times a month)  3) severe (2 times a week or more) |

The rationale for reducing the categories to three stems from two reasons. Firstly, anticipating that the five-stage categorization, made by KCHS, might be difficult to intuitively comprehend, the decision was made to reduce it to three-stage in order to facilitate understanding. Secondly, the five-stage categorization was considered to have an insufficient academic basis. In other words, since it was not developed in accordance with international standards developed by various organizations, including Centers for Disease Control and Prevention(CDC), National Institute on Alcohol Abuse and Alcoholism(NIAAA), World Health Organization(WHO), it was deemed unnecessary to adhere to the five-stage categorization. Consequently, a simpler three-stage categorization was adopted. The international standards for binge drinking, drinking status, and alcohol risk levels are listed below.

| International standards of binge drinking, drinking status, and alcohol risk levels |
| --- |
| ◼ Binge drinking^2^  🞟 Centers for Disease Control and Prevention(CDC) definition:  - more than 4 and 3 drinks on an occasion in the past 30 days for men and women, respectively.  🞟 National Institute on Alcohol Abuse and Alcoholism(NIAAA) definition:  - more than 4 and 3 drinks in a two hour period in the past 30 days for men and women, respectively.  ◼ Drinking status (CDC definition)^2^  1) infrequent (<12 drinks/year)  2) light (≥12 drinks/year to ≤3 drinks/ week)  3) moderate (4-14 and 4-7 drinks/week for men and women, respectively)  4) heavy (≥15 and ≥ 8 drinks/week for men and women, respectively)  ◼ Drinking status (NIAAA definition)^3^   \|  \| For males \| For females \| \| --- \| --- \| --- \| \| Moderate drinking \| ≤2 drinks/day \| ≤1 drinks/day \| \| Low-risk drinking \| ≤4 drinks/day or  ≤14 drinks/week \| ≤3 drinks/day or  ≤7 drinks/week \| \| Heavy drinking \| Binge drinking on 5+ days in the past 30 days \| \|   ◼ World Health Organization(WHO) Alcohol Risk Levels^4^   \|  \| World Health Organization Alcohol Risk Levels (for males) \| \| \| \| \| --- \| --- \| --- \| --- \| --- \| \| Low risk \| Medium risk \| High risk \| Very High risk \| \| Drinks per day \| 0-2.9 \| 3.0-4.3 \| 4.4-7.1 \| >7.2 \| \|  \| World Health Organization Alcohol Risk Levels (for females) \| \| \| \| \| Low risk \| Medium risk \| High risk \| Very High risk \| \| Drinks per day \| 0-1.4 \| 1.5-2.8 \| 2.9-4.3 \| >4.4 \| |

In summary, the survey questions used in our study(KCHS) did not refer to international standards for drinking. That is, it is possible to say that the survey questions may lack academic basis. This is clearly a limitation of our study. If KCHS had designed the survey questions based on international drinking status criteria, the results of our study could have been compared with other studies. However, this was not feasible in our study. We hope that in the future, KCHS will consider revising their survey questions based on international standards.

**Table 3. Homogeneity of odds ratios for suicide ideation and attempt across strata of drinking frequency and amount.**

|  | Suicide ideation | | Suicide attempt | |
| --- | --- | --- | --- | --- |
|  | Frequency of drinking | Amount of drinking | Frequency of drinking | Amount of drinking |
| p-value | 0.128 | 0.161 | 0.477 | 0.298 |

**References**

1 American Psychiatric Association. *Diagnostic and statistical manual of mental disorders : DSM-5*. 5th edition. edn, (American Psychiatric Publishing, a division of American Psychiatric Association, 2013).

2 National Center for Health Statistics. Adult Alcohol Use Information, Available from https://www.cdc.gov/nchs/nhis/alcohol/alcohol_glossary.htm. (2018).

3 National Institute on Alcohol Abuse and Alcoholism. Drinking Levels, Available from https://www.niaaa.nih.gov/alcohol-health/overview-alcohol-consumption/moderate-binge-drinking. (2023).

4 World Health Organization (WHO). International guide for monitoring alcohol consumption and related harm. (2000).
